# Serum paraoxonase activity and protein thiols in chronic renal failure patients

**DOI:** 10.4103/0971-4065.41282

**Published:** 2008-01

**Authors:** M. Prakash, J. K. Shetty, L. Rao, S. Sharma, A. Rodrigues, R. Prabhu

**Affiliations:** Department of Biochemistry, Kasturba Medical College, Manipal, India; 1 Department of Nephrology, Kasturba Medical College, Manipal, India

**Keywords:** Chronic renal failure, lipid hydroperoxides, lipid profile, oxidative stress, paraoxonase, protein thiols

## Abstract

Serum paraoxonase is known to prevent low-density lipoprotein oxidation and atherogenesis. Association of paraoxonase with the oxidative status and lipid profile in chronic renal failure (CRF) patients on conservative management and those on chronic maintenance hemodialysis was analyzed in the present study. Serum paraoxonase, protein thiols, lipid hydroperoxides, lipid profile, creatinine and albumin levels were estimated by spectrophotometric methods in CRF patients on conservative management, those on hemodialysis and in healthy controls. Total cholesterol, triglycerides, low-density lipoprotein cholesterol, lipid hydroperoxides and creatinine levels were higher and high-density lipoprotein cholesterol, protein thiols, albumin levels and paraoxonase activity were lower in patients than in healthy controls. Paraoxonase activity correlated positively with protein thiols and high-density lipoprotein cholesterol and negatively with low-density lipoprotein cholesterol and lipid hydroperoxides. In conclusion, paraoxonase activity is decreased in CRF patients particularly on chronic maintenance hemodialysis and correlates well with the oxidative stress markers.

## Introduction

Human serum paraoxonase is a calcium-dependent esterase that hydrolyzes organophosphates such as paraoxone, diazoxon, serin and soman and also arylesters such as phenyl acetate.^1^ Serum paraoxonase is a high-density lipoprotein (HDL)-associated enzyme synthesized mainly in the liver. Although the natural substrates for paraoxonase are unknown, recent studies suggest that paraoxonase prevents low-density lipoprotein (LDL) oxidation by hydrolyzing lipid peroxides.^2^ The biological role of HDL is attributed to the paraoxonase associated with it.^1^ A previous study suggests that the active site of paraoxonase contains free thiol (-SH) group present in cysteine-283. This free -SH group is believed to donate reducing equivalent to paraoxonase conferring to it a reducing property.^3^ Recent studies indicate that some other component of HDL other than free -SH of cysteine-283 in paraoxonase is responsible for the biological actions of HDL.^4^

The liver plays a key role in the synthesis of serum paraoxonase; the serum paraoxonase levels decrease in chronic liver disease.^5^ A recent study indicated the possible relation between protein-SH (protein thiol) and paraoxonase activity in alcoholic liver disease.^6^ Hepatocytes synthesize albumin, which is a major plasma protein. The -SH groups present on the protein are the major antioxidants *in vivo*. The serum levels of protein-SH in the body indicate antioxidant status and low levels of protein-SH correlated with the increased levels of advanced oxidation protein products.^7^

Patients with chronic renal failure (CRF) are at increased risk for atherosclerosis.^8^ Impaired lipoprotein metabolism was found in uremic patients and one of the abnormalities is decreased HDL cholesterol.^9^ It was found that the paraoxonase activity was significantly reduced in hemodialysis patients compared to healthy controls.^10^,^11^ Our present study aimed to investigate the relationship between serum paraoxonase activity and oxidative stress markers such as protein-SH and lipid hydroperoxides and with lipid profile parameters.

## Materials and Methods

### Subjects

The study was carried out on CRF patients on chronic maintenance hemodialysis (n = 48), CRF patients on conservative management (n = 41) and age-matched control (n = 41). The mean age of hemodialysis patients was 58 ± 7 years; that for CRF cases on conservative management was 53 ± 9 years; and that for healthy controls was 49 ± 10 years. The mean duration of hemodialysis was 16 ± 4 months. Informed consent was obtained from all subjects and the study was approved by institutional review board. Patients with liver disease were excluded from the study. None of the patients or controls received any antioxidant medication.

Under aseptic conditions, blood samples (5 ml) were drawn into plain iron-free vacutainers from antecubital veins of controls, CRF patients on conservative management and from arteriovenous fistula of hemodialysis patients immediately before initiating hemodialysis. The collected blood was allowed to clot and then centrifuged at 2000 *g* for 15 min for a clear separation of serum. All assays were performed immediately after separating the serum.

Appropriate chemicals such as paraoxone, dithionitrobenzoic acid and xylenol orange were obtained from Sigma chemicals (St Louis, MO, USA). All other reagents obtained were of analytical grade.

### Determination of paraoxonase activity

Paraoxonase activity was estimated spectrophotometrically using a method described elsewhere with minimal modifications.^10^ Briefly, the assay mixture consisted of 500 μl of 2.2 mM paraoxone substrate in 0.1 M Tris-HCl buffer, pH 8.0 containing 2 mM CaCl_2_ and 50 μl of fresh serum specimen. The absorbance was monitored at 405 nm at 25°C. One unit (IU) of paraoxonase activity is defined as 1 μmol of *p*-nitrophenol formed per min per liter at 25°C and the activity was expressed as U/L of serum.

### Determination of oxidative status

Serum protein-SH were measured by a spectrophotometric method using 5',5'-dithio-bis-2-nitrobenzoic acid^12^ and the lipid hydroperoxide content of the total serum was determined with the ferrous oxidation of xylenol orange version II assay.^13^,^14^

Fasting lipid profile was estimated by enzymatic kinetic assay method using an automated analyzer (Hitachi 912, Roche Diagnostics, Germany). Total cholesterol estimation was performed by cholesterol oxidase method; HDL cholesterol was estimated by the same method after precipitating the LDL, VLDL and chylomicrons.^15^ Triglycerides were estimated using an enzymatic mixture containing lipoprotein lipase, glycerol kinase, glycerol-3-phosphate oxidase and peroxidase.^16^ Low-density lipoprotein levels were calculated by using Friedewald's formula. Serum creatinine was estimated by spectrophotometric method using the automated analyzer.

### Statistical analysis

Results were expressed as mean ± SD. A *P* value of <0.05 was considered statistically significant. Analysis of variance was used to compare values in the three groups, followed by multiple comparisons by post-hoc test. Pearson correlation test was applied to analyze correlation between various parameters.

## Results

Mean creatinine levels in CRF patients on conservative management were 3.7 ± 1.6 mg/dL and those in CRF patients on hemodialysis were 5.2 ± 2.2 mg/dL. As depicted in Table 1, serum lipid hydroperoxides, LDL cholesterol, triglycerides and total cholesterol levels were significantly higher and HDL cholesterol levels were lower in CRF patients on conservative management and those on hemodialysis in comparison to healthy controls (*P* < 0.01). Serum paraoxonase activity was significantly reduced in CRF patients on hemodialysis compared to those on conservative management and healthy controls (*P* < 0.01); a significant decrease was also observed in paraoxonase activity in CRF patients on conservative management when compared to healthy controls (*P* < 0.01). Serum protein thiol and albumin levels were significantly lower in both CRF patients on conservative management and those on hemodialysis when compared to healthy controls (*P* < 0.01).

**Table 1:** Serum paraoxonase activity, protein thiols, albumin, lipid hydroperoxides and lipid profile in healthy controls, chronic renal failure patients on conservative management and those on hemodialysis

	Healthy controls (n = 41)	CRF cases (n = 41)	CRF cases on hemodialysis (n = 48)
Triglycerides (mg/dl)	121.12 ± 68.86	185.60 ± 101.97*	100.58 ± 47.61*
Total cholesterol (mg/dl)	159.29 ± 32.71	179.95 ± 41.45*	152.77 ± 39.55*
High-density lipoprotein cholesterol (mg/dl)	51.90 ± 15.91	42.53 ± 15.32*	45.65 ± 14.71*
Low-density lipoprotein cholesterol (mg/dl)	90.97 ± 27.53	105.18 ± 36.44*	145.32 ± 42.12*•
Paraoxonase activity (U/L)	192.53 ± 31.26	88.68 ± 38.92*	59.97 ± 36.54*•
Protein thiols (μmol/L)	342.34 ± 43.43	191.26 ± 16.75*	164.12 ± 13.54*•
Lipid hydroperoxides (μmol/L)	0.18 ± 0.09	1.78 ± 0.46*	2.17 ± 0.45*
Albumin (g/dl)	4.4 ± 1.3	2.1 ± 1.0*	2.8 ± 1.2*

* *P* < 0.01 compared to healthy controls,

• *P* < 0.01 compared to CRF cases on conservative management, CRF - Chronic renal failure

On applying the Pearson correlation test, serum paraoxonase activity correlated positively with protein thiols (*P* < 0.01), HDL cholesterol (*P* < 0.01) and negatively with lipid hydroperoxides (*P* < 0.01).

## Discussion

We observed significantly decreased paraoxonase activity in CRF patients, particularly those on chronic maintenance hemodialysis. Similar findings were obtained in hemodialysis patients by previous authors.^10^,^17^ Decrease in paraoxonase activity is associated with altered lipoprotein metabolism that favors atherogenesis with significant decrease in HDL cholesterol and increase in LDL cholesterol levels. Serum paraoxonase activity correlated positively with HDL cholesterol, indicating its association with HDL. Although several studies have proposed the antioxidative and antiatherogenic nature of paraoxonase but the exact mechanism is still not clear.

Recently, Jakubowski *et al.*, reported the natural substrate and biological role of paraoxonase.^18^ Their study suggests that paraoxonase may be involved in hydrolysis of homocysteine thiolactone into homocysteine (homocysteine thiolactonase activity). Homocysteine thiolactone is unstable and can bind to lysine residues on proteins. This *N*-homocysteinylation of proteins alters the protein structure and increased susceptibility to proteolysis. *N*-homocysteinylation of paraoxonase (or other component of HDL regulating its activity, such as apolipoprotein A1) decreases its activity. Thus, decrease in paraoxonase activity may initiate a positive feedback mechanism since a reduced paraoxonase activity will cause further accumulation of homocysteine thiolactone and may augment protein homocysteinylation.^18^–^20^

In our study, we observed a significant decrease in protein-SH levels and they correlated positively with the decrease in paraoxonase activity and albumin levels. Previous study conducted by Prakash *et al.*, demonstrated decreased protein-SH level in CRF patients on chronic maintenance hemodialysis; they also demonstrated positive association between protein-SH and albumin.^21^ Albumin is a major plasma protein and contributes greatly to plasma protein-SH pool. Homocysteinylation of albumin at cysteine-34 may make it increasingly susceptible to oxidative degradation. Increased homocysteinylation may also be responsible for decreased paraoxonase activity in CRF patients. Since paraoxonase activity is responsible for hydrolyzing homocysteine thiolactone into homocysteine, the decreased activity of paraoxonase may be the possible cause for increased protein homocysteinylation and oxidative degradation of plasma proteins, leading to decrease in available -SH groups. This may explain the possible cause for positive correlation between protein-SH and paraoxonase activity in CRF patients. Serum paraoxonase correlated positively with antioxidant protein-SH and negatively with oxidant lipid hydroperoxides, indicating itself as an oxidative stress marker.

In conclusion, serum paraoxonase activity decreases in CRF patients, particularly those on chronic maintenance hemodialysis. Serum paraoxonase activity correlated positively with protein-SH and HDL cholesterol.
